# Opening the Doors of a Substance Use Disorder Ward—Benefits and Challenges From a Consumer Perspective

**DOI:** 10.3389/fpsyt.2020.580885

**Published:** 2020-09-24

**Authors:** Regine Steinauer, Jana S. Krückl, Julian Moeller, Marc Vogel, Gerhard A. Wiesbeck, Marc Walter, Undine E. Lang, Christian G. Huber

**Affiliations:** ^1^Universitäre Psychiatrische Kliniken Basel (UPK), Klinik für Erwachsene, Universität Basel, Basel, Switzerland; ^2^Department of Psychology, Division of Clinical Psychology and Epidemiology, University of Basel, Basel, Switzerland

**Keywords:** open doors, locked ward, safety, substance use disorder, qualitative content analysis

## Abstract

Open doors in psychiatry have been a subject of controversy in recent years. While some studies postulate the clinical necessity of closed doors, others challenge the theoretical advantages of this setting, mention numerous drawbacks of closed wards, and focus on the advantages of open-door settings. With regard to patients diagnosed with substance use disorders (SUD), other standards may apply. Very little research has been done on this topic. Some studies adopted a consumer perspective (i.e. asking involved parties about their experience of the door status). To the authors’ knowledge, no study has so far addressed the ideal setting for the treatment of SUD. With our data from the opening of a specialized SUD ward, we take one step to closing this knowledge gap. Applying a qualitative design, we asked patients and health care professionals (HCP) to report changes following the opening of the ward. The results are mainly in line with the literature on the general psychiatric population. The newly introduced open-door setting was mostly perceived as positive, but some disadvantages were mentioned (e.g. less protection of patients, less control over who enters/leaves the ward, the theoretically increased risk of patients absconding). Moreover, HCP (but not patients) mentioned potentially increased substance use on the ward as an additional disadvantage that could arise. Opening a previously closed ward was generally perceived as a positive and progressive decision. These findings support the trend towards an overall open-door policy in psychiatry.

## Introduction

Opinions about closed wards in acute psychiatry vary widely. Advocates highlight the therapeutic necessity and protective atmosphere, opponents point out the ethically questionable nature of this treatment environment. The most frequently mentioned argument for closing psychiatric wards is safety (1, 2). By closing wards, health care professionals (HCP) maintain maximum control, and, therefore, may theoretically prevent patients from absconding and/or harming themselves or others (1, 2). On the other hand, closed doors impose major restrictions on the patients’ lives. Balancing patients’ safety against their autonomy is an issue that is not easily resolved (3).

This perspective paper provides the authors’ view on the question of open versus closed wards, with a focus on wards specializing in the treatment of patients with substance use disorders (SUD). Following an overview of the open-door discussion in general psychiatry, the authors explore if there are specific aspects in wards focusing on SUD. They examine the clinically important but understudied consumer perspective on less restrictive approaches in SUD inpatient treatment. In addition to consulting published literature, the authors share their own data on consumer-perspective experiences following the opening of a specialized SUD ward. Based on this information, they discuss their views on the challenges facing clinicians who wish to facilitate open-door concepts for the treatment of SUD.

### The Pros and Cons of Closed Doors in Psychiatry

The hypothesis of increased security on closed wards is supported by individual studies. Nijman et al. (4) found 30% fewer incidents of absconding on acute psychiatric units where the doors were closed for the entire shift compared to other units. Furthermore, patients and HCP report several advantages of locked wards, such as protection from third-party interference (e.g. unwanted visitors, the introduction of substances), more time for HCP to spend with patients and protection of the community (5). On the other hand, patients on locked wards feel less autonomous and are less satisfied with the treatment than patients on open wards (6, 7). They report increased feelings of boredom, depression, anxiety and frustration (2, 3, 8). Moreover, closing wards seems to increase the rate of medication refusal (9), impair therapeutic alliance (10), and also reinforce the stigma surrounding mental illness (5, 11–13).

In addition, some of the advantages postulated for locked doors are not unequivocal (14). Huber et al. (15) found that being treated in a hospital without locked wards was not associated with an increased probability of absconding, suicide, or suicide attempts. On the contrary, the authors reported a reduced risk of attempted suicide and absconding when patients were treated on an open ward. Closed wards therefore do not seem to effectively prevent patients from leaving the ward and may even increase a patient’s intention to abscond. Furthermore, it seems that imposing restrictions on patients (i.e. treatment on a closed ward) may increase violent behavior (16, 17). However, further research is needed (3, 18).

### The Pros and Cons of Closed Doors in Substance Use Disorder Wards

As to the treatment of patients with SUD, the requirements may be different than for general psychiatric wards (19). Following the American Psychiatric Association guidelines, treatment on closed wards may be advised for some patients with SUD, in particular those with reduced impulse control and/or a co-morbid psychiatric disorder which requires treatment on a closed ward (20). Another rationale may be to stop people from bringing psychoactive substances onto the ward. On the other hand, it is essential that patients with SUD learn how to deal with drug cue-related stimuli in real life. Therefore, it seems important that these issues are addressed during the hospital stay, which may possibly work best on an open ward. In addition, substance use seems to be associated with violent behavior. Elbogen et al. (21), for example, found that a mental illness with a comorbid SUD (but not a mental illness on its own) increased future violent behavior significantly. Few studies have investigated this specific sample of patients with SUD to date. However, the existing studies all favor a voluntary approach and open doors in acute psychiatric treatment of SUD (22, 23).

### Consumer Perspectives on Closed Doors in General Psychiatry

Important insights come from studies adopting a “consumer perspective”, i.e. asking patients, HCP and/or ward visitors about their perception of being on an open versus a closed ward. This perspective offers novel information about how the door status is perceived by the parties concerned. Middelboe et al. (6) reported that perceived ward atmosphere predicts patients’ satisfaction which, in turn, is associated with treatment compliance and outcome variables (24–27).

#### Advantages of Closed Doors

Patients on general psychiatric wards confirm the frequently stated—though not uncontroversial—advantages of closed doors concerning safety. Patients report an increased sense of safety due to the HCP’s greater control over patients and better protection against outside influences (28, 29), as well as less absconding and less aggression towards the general public (30). Patients also report that closed doors enable HCP to have more time for patients and promote secure and efficient care; this in turn makes patients feel safe and calm (30). HCP report comparable advantages of closed wards (8, 28, 30). Staff members mention that less staff and less close observation is needed when doors are closed (8, 28). Moreover, according to HCP, there is more contact with patients and visitors, facilitating monitoring (8, 28). Bowers et al. (30) indicate reduced anxiety and a greater sense of control and confidence reported by HCP. Patients, HCP and visitors share the perception that closing ward doors may reduce absconding (3).

#### Disadvantages of Closed Doors

Patients’ concerns mainly focus on adverse effects on their emotional condition, i.e. feeling confined, dependent and frustrated (29, 30). A non-caring closed-ward environment may foster greater authoritarianism in a cold milieu (30) and patients’ passiveness (29). Moreover, patients may need to adapt to other patients’ needs (28, 29). Patients also perceive greater power of staff members (29). HCP report similar disadvantages of closed doors (8, 28, 30). In addition, HCP mention a higher workload on closed wards and more effort to explain why the door is locked (8). Patients’ feelings of confinement are sometimes confirmed by staff members, e.g. HCP report a more volatile environment (28) and a sense of being locked in/being unsafe (8).

In conclusion, all consumer parties report possible positive as well as negative aspects of locked doors. Patients, especially, have mixed feelings about the door status (3). Reports are sometimes inconclusive—e.g. HCP attribute a higher or lower workload to closed-door settings. In particular, there is still a considerable knowledge gap concerning consumer perspectives on open vs. locked doors in SUD wards.

## Consumer Perspectives on Closed Doors in Substance Use Disorder Wards: Case Study on the Opening of a Specialized SUD Ward

To the authors’ knowledge, no study to date has addressed a consumer perspective in an SUD sample. Thus, in addition to existing literature, this perspective paper explores the experiences of patients, HCP on an open (formerly closed) SUD ward.

### Setting

The ward studied specializes in the treatment of patients with SUD, in particular with an alcohol or drug dependence syndrome. The main focus of the ward lies on qualified detoxification treatment, including diagnosis and treatment of concomitant mental and somatic diseases. The unit had a capacity of 13 inpatients at the time of the investigation. It was opened in July 2011, and data was collected in May 2012. The door was open from both sides during daytime, at nighttime the door was closed.

All patients treated in the open unit at the time of the survey who had previously been hospitalized in the unit at least once prior to the opening were asked to participate in the study (inclusion criteria). One patient refused to participate. In addition, the interdisciplinary HCP team was asked to give written feedback and to attend a moderated focus group to discuss the changes experienced since the opening of the ward. Mixed (oral and written) data collection was chosen to get as many opinions as possible. All staff members had the opportunity to express their opinion regardless of whether they participated in the focus group. The interdisciplinary team consisted of psychiatric nurses, physicians, psychologists and social workers. Oral informed consent was obtained from all participants. The participation of patients and HCP was voluntary, none of the participants was compensated financially or otherwise, and non-participation had no adverse consequences.

Patients who met the inclusion criteria and gave informed consent were invited to the semi-structured interview. During the interview, patients were asked open questions about several topics, e.g. general changes since the opening of the ward, well-being, ward atmosphere, relationship to HCP, feeling of safety, personal responsibility, and freedom. Based on the patient’s answers, the interviewer asked follow-up questions to explore more specific aspects. The narrative interviews were held in German. The duration was approximately 30 min per participant.

Our approach was based on an inductive qualitative study design. The interviews and the discussion in the focus group were recorded, transcribed and evaluated anonymously. We analyzed all the data, applying qualitative content analysis as described by Mayring (31). This qualitative approach is a step-by-step formulation of inductive categories as close as possible to the material. Within a feedback loop, those categories are revised, reduced to main categories and checked in respect to their reliability. The results were validated by discussing them with other patients in the same ward at a later time. The main results were presented in a therapeutic group setting asking for critical feedback. All results were confirmed. Some of this work was presented at the “9. Dreiländerkongress Pflege in der Psychiatrie” in Vienna, Austria, and an abstract was published in the accompanying conference proceedings (32). Data and analyses for the current paper were collected as part of clinical quality management during the transition from the closed to the open ward period. Scientific analysis and publication were not planned at that time. However, the local ethics committee gave retrospective approval that the study is in agreement with the ethical guidelines according to the Human Research Act art. 51 par. 2.

### Limitations

Data for this investigation were collected as part of clinical quality management. Whereas this, in theory, could have impaired data quality, scientifically appropriate methods were used for data acquisition and analysis. The current study examined a limited patient and HCP sample: only patients who were treated during the time of assessment and who had also been patients previously (while the locked-door policy was in place) were eligible for inclusion, and only HCP from the ward where the open-door policy was implemented could give feedback. The limited sample certainly impairs generalizability of the results. In addition, selection bias arising from choosing a convenience sample cannot be ruled out. The patient sample in particular may have been highly selective, as patients supporting the open-door policy possibly showed more interest in participating in the study. On the other hand, all members of the HCP team gave feedback on their impression of the changes, ensuring a rather complete picture of the study group. Moreover, it is to be kept in mind that qualitative studies commonly have a hypothesis-generating nature and report personal experiences (rather than providing proof). The analyses presented are based on data acquired in 2012 and are relatively old. However, it seems unlikely that the improvements and challenges present during the transition to an open-ward setting have changed relevantly in 2020. Given the scant literature available on the topic, the authors are convinced that the results are still of interest and will be clinically useful for HCP who are in the process of taking on the challenge of opening an SUD specific ward today.

### Results

Five patients agreed to provide feedback on their perspective. Relevant demographic characteristics of these participants are shown in **Table 1**.

**Table 1:** Demographic features of patient participants.

	Age range (in years)	Most recent inpatient treatment	Number of inpatient stays (total)
**P_1**	35–39	2011	2
**P_2**	40–44	2011	4
**P_3**	50–54	2011	3
**P_4**	45–49	2010	2
**P_5**	35–39	2010	14

To present information on demographic characteristics of the patients without endangering pseudonymization, age is given in 5-year intervals, and the year of the most recent inpatient treatment and total number of inpatient stays are provided. P_number, pseudonym of the cited patient.

All 18 staff members stated possible advantages and disadvantages and included daily experiences at the open unit in written feedback. In addition, eight psychiatric nurses as well as a senior physician and a psychologist participated in the group discussion.

Changes following the transition to an open-door treatment setting reported by patients and HCP were categorized using qualitative content analysis and are presented in **Table 2**.

**Table 2:** Changes after transformation from a closed ward to an open ward as reported by patients and health care personnel, categorized using qualitative content analysis according to Mayring (30).

Category	Quotes
Time	“All in all you notice that the matters have calmed down … It’s calmer now … You can notice it with personnel, too… You have more time.” (P_2)
Relationship	“Building relationships positive due to less control functions” (TEAM) “It is a cooperative contact [note: with the HCP]…” (P_3)
Personal Responsibility	“As we put more trust in the patients, they assume more responsibility” (TEAM) “It’s a goal to achieve more responsibility [note: in the patient]” (P_2)
Well-being	“Just the feeling that you can simply enter … and to know that the door is open … this was a good feeling” (P_4) “For the patient, it’s much more pleasant.” (P_2)
Treatment	“Now, due to the open environment, you can focus on other important tasks” (TEAM) “Closed wards make me nervous [… ] It’s like in prison” (P_1) “Everything seems less military” (P_2)
Aggression	“In my opinion, the closed environment does indeed promote aggression…”(P_3)
Destigmatization	“When there are visitors, they were shocked … My brother visited with his children, and they asked: what is it, why are the doors locked?” (P_1)
Exchange	“You tend to reflect on what could be good for me … Before, in the closed environment, everyone was just fighting for herself … for their own rights and liberties.” (P_5)
Protection	“A few patients need the feeling of safety because of locked doors.” (TEAM)
Overview	“Whereabouts of certain patients sometimes unclear” (TEAM)
Risk of absconding	“There are patients who are suddenly leaving and running away.” (P_5)
Substance use^1^	“No more substance use in secret” (TEAM)

Sample quotes for the identified categories were translated from German and are cited in the right column. P: quote by one of the interviewed patients; P_number: pseudonym of the cited patient; TEAM: quote by a member of health care personnel (HCP). ^1^Only mentioned by HCP.

The positive aspects that patients as well as HCP mentioned after the introduction of the open-door policy included the following: there was more time for HCP to address patients’ needs and therapeutic contacts, the therapeutic relationship improved, patients received and assumed more personal responsibility, an atmosphere of trust emerged, and well-being increased for patients and HCP. It was also mentioned that the open setting served to de-escalate aggression, and that the new setting was found to be less stigmatizing. HCP reported that there was less clandestine substance use on the ward.

However, patients and HCP also reported some negative aspects, e.g. that some patients felt less protected, that it was more difficult to know who was on the ward and who had left, and that some patients left the ward without previous discussion. HCP, but not patients, reported that substance use on the ward could theoretically increase after the introduction of the open-door setting.

## Benefits and Challenges of Opening the Doors of an SUD Ward

In our case study, patients of an inpatient unit specializing in the acute psychiatric treatment of SUD were interviewed about the changes following the opening of a previously closed ward. The HCP team gave written feedback on possible benefits and implications of the opening. In a moderated focus group, changes experienced since the opening of the unit were discussed. The changes reported by patients were very similar to the ones stated by HCP. Both groups reported mostly positive effects of the opening. Reduced protection, impaired overview of the ward, an increased risk of absconding, and potentially increased substance use were identified as possible disadvantages of unlocked doors. Especially the disadvantage of reduced protection has been reported by other research groups who investigated mainly quantitative data (3, 8, 28, 29). Despite some limitations, the findings of our qualitative study seem to be in line with previous research.

Unlocking the doors changed the general attitude of HCP in how they approached their patients, as reported by patients and HCP alike. This was indicated in particular by answers from the categories “time”, “personal responsibility” and “treatment”. This finding is mostly in accordance with the literature (8, 29). In the study by Muir-Cochrane et al. (3), locked doors were mentioned as a symbol of mistrust, indicating the negative influence of the door status on the HCP-patient relationship. On the other hand, in contrast to recent studies (3, 28, 29), our participants experienced staff having more time for patients during the open compared to the closed ward period. The main reason mentioned for this observation in our sample was having less “control functions” (i.e. opening and closing the doors, explaining why the door is closed) on the open ward. The HCP had a reduced sense of being “the guard with the keys”. Another aspect mentioned by other researchers (3, 28) was the difficulty of maintaining an overview on an open ward. Keeping track of the whereabouts of patients may lead to an “anxious vigilance” (3) which represents an additional task and possibly leads to stress. Nonetheless, our HCP experienced having more time after the ward was opened. Haglund et al. (8, 29) discussed the aspect that family members might feel more supported by the protecting environment of a closed unit. This point was not raised by patients and HCP in our case study, but both groups mentioned that family members had the impression that the novel treatment setting was less stigmatizing. This finding is supported by Ashmore (28).

Investigating an SUD sample, illness-specific aspects like substance use were mentioned. Interestingly, a reduction of clandestine substance use on the ward was only stated by HCP. The staff perceived patients being more open about their substance use during the hospital stay. Simpson et al. (33) did not report a consistent relationship between substance use and exit security features, intensity of drug/alcohol monitoring and the locking of the ward door. In another study, our research group found no relevant change of substance use on a specialized SUD and dual-diagnosis ward (not the ward under study here) comparing the time period when the ward was closed to the period when the same ward was open (34). As substance use was screened for by urinalysis and breathalyzer testing in the study mentioned above, there may indeed be no increase in substance use after introduction of an open-door policy. The hypothesis that opening an SUD ward might increase substance use may be unfounded, but the findings still have to be replicated in future research.

Open-door policies have been associated with an improved ward atmosphere (7, 35–37). Patients and HCP in our study reported improved well-being and improved establishment of therapeutic relationships due to decreasing control functions after opening the ward. This corresponds with the literature, as higher general satisfaction with treatment on open units has already been found by Müller et al. (7) and Middelboe et al. (6). In addition, the impression of reduced aggression on open wards was reported in our sample. This is also supported by several studies which investigated various wards and were based on objective measures (6, 30, 38).

### Conclusion

The discussion about opening or locking doors in psychiatry is a clinically relevant, but highly controversial topic. Depending on the patient’s characteristics (e.g. diagnosis, co-morbid disorders, violent behavior), one of the two treatment settings might be preferred in daily clinical practice. However, the literature about the door status in general psychiatry clearly favors open wards. Less research is available on SUD populations, but this still favors an open-door approach. As to the consumer perspective, the picture is similar for general psychiatry wards (also favoring open wards), but there is little research and the existing literature is partly inconclusive (e.g. lower or higher workload on closed wards?). Our small set of data mainly suggests that these findings may also be valid for the SUD population, but we certainly do not claim generalizability. Further research is recommended. Altogether, strong advantages of open doors seem to outweigh the frequently cited disadvantages. We therefore encourage clinicians to take bold steps towards an overall open-door policy in psychiatry.

## Data Availability Statement

The raw data supporting the conclusions of this article will be made available by the authors, without undue reservation.

## Ethics Statement

The studies involving human participants were reviewed and approved by Ethikkommission Nordwest- und Zentralschweiz (Hebelstrasse 53, 4056 Basel; www.eknz.ch, tel. 061 268 13 50, fax 061 268 13 51, email: eknz@bs.ch). Written informed consent for participation was not required for this study in accordance with the national legislation and the institutional requirements.

## Author Contributions

RS designed the study. RS collected the data. RS analyzed and interpreted the data. RS and CH wrote the initial draft of the paper. JK, JM, MV, GW, MW, and UL revised the manuscript for important intellectual content. RS had full access to all the data in the study and takes responsibility for the integrity of the data and the accuracy of the data analysis. All authors contributed to the article and approved the submitted version.

## Conflict of Interest

The authors declare that the research was conducted in the absence of any commercial or financial relationships that could be construed as a potential conflict of interest.
